# Abiraterone or Enzalutamide for Patients With Metastatic Castration-Sensitive Prostate Cancer: A Nationwide Veterans Affairs Study

**DOI:** 10.1200/CCI-25-00213

**Published:** 2026-07-16

**Authors:** Jennifer La, Lin Wang, June K. Corrigan, Deborah Lang, Nhan V. Do, Mary T. Brophy, Nathanael R. Fillmore, Channing J. Paller

**Affiliations:** ^1^ VA Cooperative Studies Program, VA Boston Healthcare System, Boston, MA; ^2^ Department of Medicine, Harvard Medical School, Boston, MA; ^3^ Department of Epidemiology, Johns Hopkins Bloomberg School of Public Health, Baltimore, MD; ^4^ Department of Dermatology, Boston University Chobanian & Avedisian School of Medicine, Boston, MA; ^5^ Section of General Internal Medicine, Boston University Chobanian & Avedisian School of Medicine, Boston, MA; ^6^ Section of Hematology & Medical Oncology, Boston University Chobanian & Avedisian School of Medicine, Boston, MA; ^7^ Department of Oncology, Johns Hopkins University School of Medicine, Baltimore, MD

## Abstract

**PURPOSE:**

Enzalutamide or abiraterone, combined with androgen-deprivation therapy, is standard of care for metastatic castration-sensitive prostate cancer (mCSPC). However, no trials have compared these drugs. This study compared clinical outcomes in patients with mCSPC treated with enzalutamide or abiraterone.

**METHODS:**

This retrospective cohort study included patients with mCSPC initiating enzalutamide or abiraterone between January 1, 2020, and December 31, 2023, within the nationwide US Veterans Affairs health care system. Inverse probability of treatment weighting was used to balance baseline characteristics. Restricted mean survival time (RMST) differences in overall survival (OS), time to treatment switch or death (TTS), and prostate cancer survival (PCS) were evaluated.

**RESULTS:**

The study included 5,135 patients with mCSPC treated with enzalutamide (1,803) or abiraterone (3,332). The median age was 74.33 years; 58.0% were non-Hispanic White, 28.2% non-Hispanic Black, and 5.6% Hispanic. After weighting, baseline characteristics were well balanced. The median follow-up was 18.74 months for abiraterone and 24.76 months for enzalutamide. Outcomes were similar overall: for OS, the 3-year RMST difference was 0.72 months (95% CI, -0.06 to 1.50); for TTS, the 3-year RMST difference was 0.53 months (95% CI, -0.45 to 1.51), and for PCS, the 1-year RMST difference was -0.12 months (95% CI, -0.35 to 0.11). In subgroup analysis, enzalutamide was associated with improved OS among patients age 75 years and older (3-year RMST difference: 1.65 months, 95% CI, 0.41 to 2.89), but not among younger patients (3-year RMST difference: 0.13 months, 95% CI, -0.86 to 1.11).

**CONCLUSION:**

In this nationwide cohort study, enzalutamide and abiraterone yielded comparable OS, TTS, and PCS outcomes overall, although a small but statistically significant OS benefit was observed for enzalutamide among older patients (≥75 years). These real-world findings from the largest integrated US health care system may provide guidance for selecting mCSPC treatments, although residual confounding cannot be fully excluded.

## INTRODUCTION

Clinical trials have established that androgen-receptor pathway inhibitor (ARPI) compounds, including enzalutamide or abiraterone, in combination with androgen deprivation therapy (ADT), offer benefit over ADT alone for treating metastatic castration-sensitive prostate cancer (mCSPC).^1-4^ Enzalutamide or abiraterone is approved for use in mCSPC. However, the two drugs have notable differences, including their mechanisms of action, toxicities, and costs.^5-9^ Although both drugs target the androgen-receptor signaling pathway, their mechanisms of action differ. Abiraterone inhibits the CYP17A enzyme and androgen synthesis, whereas enzalutamide blocks multiple downstream events such as androgen-receptor nuclear translocation and transcription-related processes.^10,11^ No clinical trials have compared enzalutamide versus abiraterone, and it has remained unclear whether enzalutamide or abiraterone is superior in the setting of mCSPC. Existing data based on meta-analysis of clinical trials^12-14^ and small retrospective studies^15-17^ are limited, with conflicting and nondefinitive results.

**CONTEXT**

**Key Objective**
Among patients with metastatic castration-sensitive prostate cancer (mCSPC), does enzalutamide or abiraterone yield superior outcomes? No head-to-head trials have compared these agents, leaving treatment selection uncertain.
**Knowledge Generated**
In this nationwide Veterans Affairs cohort of 5,135 patients with mCSPC, enzalutamide and abiraterone yielded comparable overall survival, time to treatment switch or death, and prostate cancer survival. A small but statistically significant advantage for enzalutamide was observed among patients age 75 years and older.
**Relevance *(Z. Bakouny)***
Enzalutamide and abiraterone have not been compared head to head in clinical trials for metastatic castrate-sensitive prostate cancer. This retrospective study finds that these two agents have roughly equivalent efficacy overall, with a potential benefit for enzalutamide over abiraterone among patients who are over 75 years of age.*
*Relevance statement written by *JCO Clinical Cancer Informatics* Associate Editor Ziad Bakouny, MD, MSc.

The purpose of the present study was to compare clinical outcomes in patients with mCSPC initiating treatment with enzalutamide or abiraterone, using data from the national US Department of Veterans Affairs (VA) health care system. We evaluated differences overall and in subgroups, using rigorous methodology with systematically defined data elements.

## METHODS

### Study Design, Data Sources, and Participants

This retrospective cohort study compared outcomes of patients with mCSPC in the VA health care system who initiated treatment with abiraterone versus enzalutamide. Data were obtained from the VA Corporate Data Warehouse (CDW), which collates administrative and electronic health record data from VA facilities throughout the United States.^18^ The study was approved by the VA Boston Research and Development Committee as an exempt study before data collection and analysis with a waiver of informed consent due to existing data usage, per the Common Rule.^19^

The study included patients with mCSPC who initiated treatment with abiraterone or enzalutamide between January 1, 2020, and December 31, 2023. January 1, 2020, was chosen as the start date because abiraterone and enzalutamide were approved for patients with mCSPC on February 7, 2018, and December 16, 2019, respectively.^20,21^ Treatments given in inpatient and outpatient settings at the VA, as well as claims for outside care paid for by the VA, were captured. Patients were said to have castration-sensitive disease if they failed to be identified as castration-resistant using a previously published and validated (97.9% sensitivity, 99.2% specificity) method before and up to 30 days after the initial treatment with abiraterone or enzalutamide.^22^ Metastatic status was obtained from radiology reports and clinical notes using a previously published and validated method.^23^ Patients were excluded if they were treated with chemotherapy (listed in Data Supplement Methods) between the metastatic date and abiraterone or enzalutamide initiation, if they lacked follow-up data, or if they lacked a baseline prostate-specific antigen (PSA) value within 365 days before the index date.

### Outcomes and Covariates

Three time-to-event outcomes were evaluated: overall survival (OS), time to treatment switching or death (TTS), and prostate cancer survival (PCS). OS was defined as the time from the index date to death from any cause, as recorded in the VA CDW. Patients were censored on December 31, 2023 (the end of the study period). Mortality data at the VA are highly accurate and complete,^24^ so it was unnecessary to censor for loss to follow-up. TTS was defined as the time from the index date to a new prostate cancer treatment (listed in Data Supplement Methods) after the initiation of abiraterone or enzalutamide, or death, whichever occurred first. Patients were censored at the end of the study period or at the first break in continuous follow-up after the index date. PCS was defined as the time from the index date to death from prostate cancer, as recorded on the death certificate from the National Death Index and linked to VA patient identifiers using the VA Mortality Data Repository. Patients were censored on the date they died from another cause or on December 31, 2021, which was the most recent date for which data were available in the Mortality Data Repository at the time of analysis.

Covariates were defined based on data recorded before the index date. Age and self-reported race/ethnicity were ascertained using structured data in the VA CDW. Frailty was measured using the VA Frailty Index,^25^ and individual comorbidities were measured using definitions from the Centers for Medicare & Medicaid Services Chronic Conditions Warehouse,^26^ based on both diagnosis and procedure codes recorded in the 3 years before the index date. Prior treatment information and laboratory test results were obtained from structured data. Detailed definitions for all covariates are in Data Supplement Methods.

### Statistical Analysis

Inverse probability of treatment weighting (IPTW) was used to control for potential confounders, including age, race/ethnicity, comorbidities, frailty, treatment initiation year, and prior treatment. The predicted probability (ie, propensity score) of a patient starting enzalutamide versus abiraterone given the patient's characteristics at baseline was estimated using multivariable logistic regression that included these potential confounders as covariates. The distribution of propensity scores was examined for overlap between the two treatment groups. The propensity scores were used to calculate stabilized weights for IPTW. Stabilized weights were examined for extreme outliers. After weighting, balance was inspected by tabulating weighted patient characteristics within the treatment groups and examining the standardized mean difference (SMD) across groups, where SMD <0.1 conventionally indicates good balance.^27^

After IPTW, Kaplan-Meier analysis was used to estimate the survival function for each outcome, stratified by treatment type. An estimation was made of restricted mean survival time (RMST), which is defined as the area under the survival curve up to specific time points. RMST differences were used to compare patients initiating enzalutamide versus abiraterone. In addition, Cox models were used to estimate time-varying hazard ratios. Analyses were also conducted in preplanned subgroups defined by PSA doubling time, age, and race. The median follow-up was determined using reverse Kaplan-Meier. Curves were truncated when <10 patients are at risk. All analyses were conducted using R software, version 4.1.2.

## RESULTS

**FIG 1.**
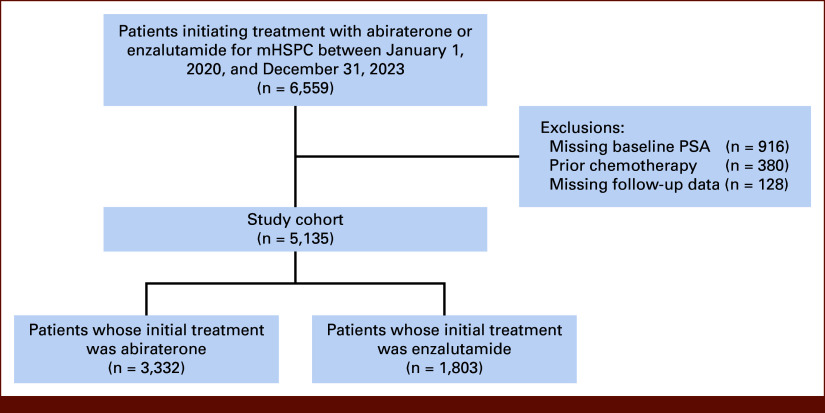
Flow diagram depicting selection of study cohort. mHSPC, metastatic hormone-sensitive prostate cancer.

We identified 5,135 patients meeting the inclusion criteria for this study (Fig 1), of whom 3,332 initiated abiraterone and 1,803 initiated enzalutamide for mCSPC. The median age was 74.33 years (IQR, 69.66 to 78.88 years); 270 (5.6%) were Hispanic; 2,978 (58.0%), non-Hispanic White; 1,446 (28.2%), non-Hispanic Black; 441 (8.6%), other or unknown. Table 1 summarizes patient characteristics before and after IPTW. Before weighting, patients treated with enzalutamide were more likely to have heart disease and diabetes. After weighting, patient characteristics were well balanced (SMD <0.1; Table 1; Data Supplement, Fig S1). There was no evidence of nonpositivity or misspecification of the propensity score model based on an examination of score distributions (Data Supplement, Figs S2 and S3), with a 1.0 mean stabilized weight and no extreme weights (minimum stabilized weight, 0.49; maximum, 2.38). For OS, the median follow-up was 18.74 months for abiraterone (IQR, 18.12 to 19.86) and 24.76 months for enzalutamide (IQR, 23.64 to 25.97). For PCS, the median follow-up was 10.19 months for abiraterone (IQR, 9.40 to 11.05) and 11.61 months for enzalutamide (IQR, 10.72 to 12.69). For TTS, the median follow-up was 10.85 months for abiraterone (IQR, 10.29 to 11.28) and 10.95 months for enzalutamide (IQR, 10.13 to 11.87).

**TABLE 1.** Patient Characteristics Before and After Inverse Probability of Treatment Weighting

Variable	Unweighted				Weighted			
	Overall	Abiraterone	Enzalutamide	SMD	Overall	Abiraterone	Enzalutamide	SMD
Patients, No.	5,135	3,332	1,803		5,132.2	3,336.3	1,795.9	
Age, median (IQR)	74.33 (69.66, 78.88)	74.22 (69.36, 78.48)	74.66 (70.28, 79.80)	0.129	74.37 (69.69, 79.01)	74.38 (69.81, 78.86)	74.31 (69.54, 79.28)	0.011
Race/Ethnicity, No. (%)				0.124				0.004
Hispanic	270 (5.3)	151 (4.5)	119 (6.6)		275.3 (5.4)	178.6 (5.4)	96.7 (5.4)	
Non-Hispanic White	2978 (58.0)	1,934 (58.0)	1,044 (57.9)		2973.4 (57.9)	1,931.6 (57.9)	1,041.8 (58.0)	
Non-Hispanic Black	1,446 (28.2)	980 (29.4)	466 (25.8)		1,439.2 (28.0)	937.6 (28.1)	501.6 (27.9)	
Other or unknown	441 (8.6)	267 (8.0)	174 (9.7)		444.3 (8.7)	288.4 (8.6)	155.8 (8.7)	
Year of initial treatment, No. (%)				0.27				0.01
2020	1,067 (20.8)	599 (18.0)	468 (26.0)		1,078.7 (21.0)	698.7 (20.9)	380.1 (21.2)	
2021	1,186 (23.1)	713 (21.4)	473 (26.2)		1,190.9 (23.2)	771.9 (23.1)	419.0 (23.3)	
2022	1,398 (27.2)	958 (28.8)	440 (24.4)		1,396.4 (27.2)	907.4 (27.2)	489.0 (27.2)	
2023	1,484 (28.9)	1,062 (31.9)	422 (23.4)		1,466.1 (28.6)	958.3 (28.7)	507.9 (28.3)	
Prior treatment, No. (%)								
Radiation therapy	502 (9.8)	325 (9.8)	177 (9.8)	0.002	495.8 (9.7)	323.3 (9.7)	172.5 (9.6)	0.003
Prostatectomy	367 (7.1)	259 (7.8)	108 (6.0)	0.07	360.8 (7.0)	237.0 (7.1)	123.8 (6.9)	0.008
Bisphosphonate	322 (6.3)	188 (5.6)	134 (7.4)	0.072	316.9 (6.2)	207.1 (6.2)	109.8 (6.1)	0.004
PSA, median (IQR)	11.10 (2.16, 57.34)	11.88 (2.33, 57.38)	9.93 (1.85, 57.26)	0.016	11.17 (2.15, 57.32)	12.40 (2.40, 58.19)	9.78 (1.71, 56.00)	0.009
PSA doubling time, median (IQR)	2.41 (-0.96, 3.25)	2.34 (-1.09, 3.12)	2.41 (-0.73, 3.41)	0.014	2.41 (-0.94, 3.19)	2.40 (-1.05, 3.10)	2.41 (-0.75, 3.36)	<0.001
Comorbidity, No. (%)								
Acute myocardial infarction	184 (3.6)	111 (3.3)	73 (4.0)	0.038	187.4 (3.7)	121.2 (3.6)	66.2 (3.7)	0.003
Atrial fibrillation	693 (13.5)	430 (12.9)	263 (14.6)	0.049	704.5 (13.7)	454.1 (13.6)	250.4 (13.9)	0.01
Cardiac arrhythmia	1,397 (27.2)	878 (26.4)	519 (28.8)	0.055	1,407.5 (27.4)	912.3 (27.3)	495.1 (27.6)	0.005
Complicated Hypertension	1,012 (19.7)	615 (18.5)	397 (22.0)	0.089	1,021.9 (19.9)	662.8 (19.9)	359.1 (20.0)	0.003
Congestive heart failure	690 (13.4)	381 (11.4)	309 (17.1)	0.164	702.1 (13.7)	454.3 (13.6)	247.8 (13.8)	0.005
Heart failure	622 (12.1)	344 (10.3)	278 (15.4)	0.153	636.1 (12.4)	411.3 (12.3)	224.8 (12.5)	0.006
Peripheral vascular disease	895 (17.4)	550 (16.5)	345 (19.1)	0.069	894.1 (17.4)	581.2 (17.4)	312.8 (17.4)	<0.001
Stroke transient ischemic attack	367 (7.1)	244 (7.3)	123 (6.8)	0.02	366.4 (7.1)	238.6 (7.2)	127.8 (7.1)	0.001
Valvular disease	405 (7.9)	246 (7.4)	159 (8.8)	0.053	416.5 (8.1)	268.2 (8.0)	148.3 (8.3)	0.008
Alzheimer's disease	489 (9.5)	315 (9.5)	174 (9.7)	0.007	487.4 (9.5)	316.8 (9.5)	170.5 (9.5)	<0.001
Anemia	1,730 (33.7)	1,078 (32.4)	652 (36.2)	0.08	1,727.7 (33.7)	1,123.8 (33.7)	603.9 (33.6)	0.001
Cerebrovascular disease	622 (12.1)	402 (12.1)	220 (12.2)	0.004	623.1 (12.1)	405.1 (12.1)	218.0 (12.1)	<0.001
Chronic kidney disease	1,869 (36.4)	1,201 (36.0)	668 (37.0)	0.021	1,877.7 (36.6)	1,219.1 (36.5)	658.6 (36.7)	0.003
Diabetes	1,858 (36.2)	1,107 (33.2)	751 (41.7)	0.175	1,866.1 (36.4)	1,210.9 (36.3)	655.2 (36.5)	0.004
Liver disease	495 (9.6)	306 (9.2)	189 (10.5)	0.044	492.8 (9.6)	320.7 (9.6)	172.0 (9.6)	0.001
Viral Hepatitis	231 (4.5)	157 (4.7)	74 (4.1)	0.03	230.2 (4.5)	149.7 (4.5)	80.4 (4.5)	<0.001
VA frailty index, median (IQR)	0.19 (0.13, 0.32)	0.19 (0.13, 0.29)	0.23 (0.13, 0.32)	0.12	0.19 (0.13, 0.32)	0.19 (0.13, 0.32)	0.19 (0.13, 0.29)	0.002
Hemoglobin, median (IQR)	13.00 (11.50, 14.20)	13.00 (11.60, 14.20)	12.80 (11.30, 14.00)	0.088	12.90 (11.50, 14.20)	13.00 (11.50, 14.20)	12.90 (11.40, 14.10)	0.031
LDH, median (IQR)	191.00 (158.00, 257.00)	189.00 (159.00, 259.00)	194.50 (157.75, 248.50)	0.09	191.20 (158.00, 258.00)	189.00 (159.35, 259.12)	195.91 (158.00, 253.61)	0.09

NOTE. High SMD values (conventionally >0.1) in the unweighted cohort indicate imbalance that may confound treatment effect estimates, whereas low SMD values after weighting indicate adequate covariate balance.

Abbreviations: LDH, lactate dehydrogenase; PSA, prostate-specific antigen; SMD, standardized mean difference.

**FIG 2.**
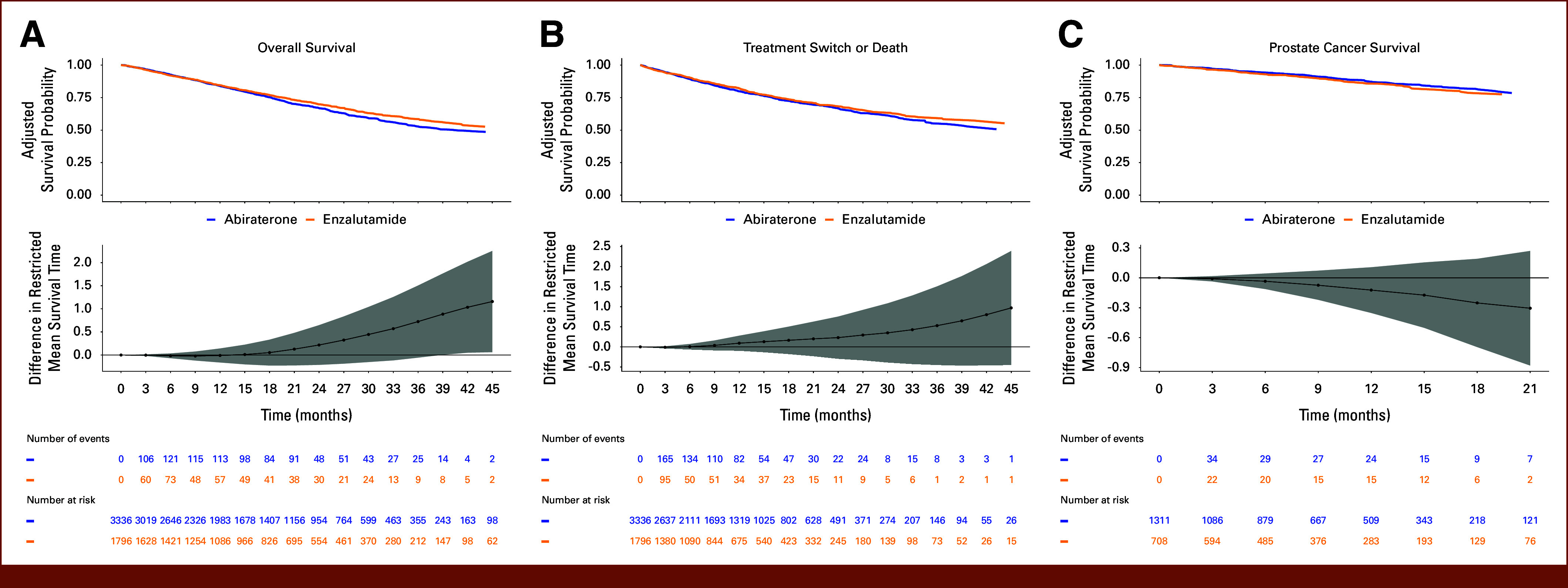
Outcomes in patients initially treated for abiraterone versus enzalutamide after IPTW. Outcomes include (A) OS, (B) TTS, and (C) PCS. In addition to Kaplan-Meier plots, the difference in RMST at each time point (3-month increments) is shown. The RMST at time t measures the mean survival censoring at t and is equal to the area under the Kaplan-Meier plot up to t, for OS, TTS, and PCS. IPTW, inverse probability of treatment weighting; OS, overall survival; PCS, prostate cancer survival; RMST, restricted mean survival time; TTS, time to treatment switch or death.

**FIG 3.**
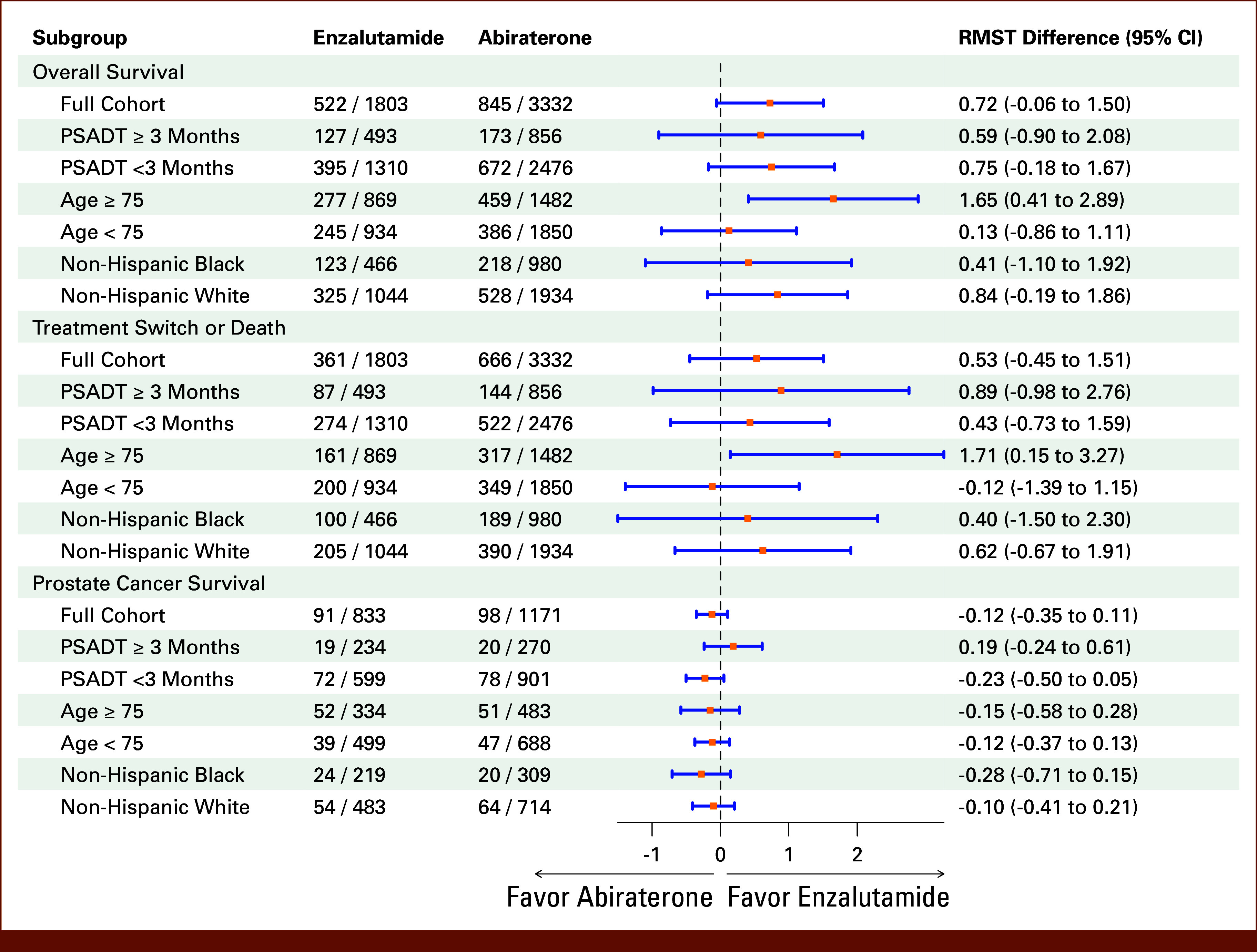
The difference in RMST, along with 95% CIs, for the full cohort and for subgroups defined by PSADT. Outcomes include OS, TTS, and PCS. RMST for OS and TTS is measured at 3 years. RMST for PCS is measured at 1 year due to availability of follow-up data. OS, overall survival; PCS, prostate cancer survival; PSA, prostate-specific antigen; PSADT, PSA doubling time; RMST, restricted mean survival time; TTS, time to treatment switch or death.

Overall, outcomes were similar between patients treated with enzalutamide or abiraterone. For OS, RMST was 27.99 months (95% CI, 27.37 to 28.61) for enzalutamide and 27.27 months (95% CI, 26.79 to 27.74) for abiraterone at 3 years, a difference of 0.72 months (95% CI, -0.06 to 1.50; Figs 2A and 3; Data Supplement, Fig S4). Similarly, for TTS, RMST was 27.34 months (95% CI, 26.56 to 28.12) for enzalutamide and 26.81 months (95% CI, 26.22 to 27.41) for abiraterone at 3 years, a difference of 0.53 months (95% CI, -0.45 to 1.51; Figs 2B and 3). Finally, for PCS, RMST was 11.18 months (95% CI, 11.00 to 11.36) for enzalutamide and 11.30 months (95% CI, 11.16 to 11.44) for abiraterone at 1 year, a difference of -0.12 months (95% CI, -0.35 to 0.11; Figs 2C and 3).

Interestingly, there was an advantage for enzalutamide for patients age 75 years and older, but not for patients younger than 75 years. Among patients age 75 years and older, the RMST for OS at 3 years was 26.47 months for enzalutamide (95% CI, 25.49 to 27.44) and 24.82 months for abiraterone (95% CI, 24.04 to 25.59), a difference of 1.65 months (95% CI, 0.41 to 2.89; Fig 3; Data Supplement, Figs S5-S6). In contrast, among patients younger than 75 years, the RMST for OS at 3 years was 29.30 months for enzalutamide (95% CI, 28.51 to 30.09) and 29.18 months for abiraterone (95% CI, 28.59 to 29.76), a difference of 0.13 months (95% CI, -0.86 to 1.11; Fig 3; Data Supplement, Figs S7 and S8). A similar pattern was observed for TTS, but not for PCS (Fig 3; Data Supplement, Figs S5-S8). In subgroups defined by PSA doubling time (≥3 months *v* <3 months) or race (non-Hispanic White *v* non-Hispanic Black), we did not observe any significant differences in RMST between enzalutamide and abiraterone for any of the outcomes (Fig 3; Data Supplement, Figs S9-S16).

## DISCUSSION

In this cohort study of patients with mCSPC in the largest integrated health care system in the United States, we found that enzalutamide and abiraterone yielded comparable OS, TTS, and PCS outcomes. There was a significant benefit for enzalutamide among older patients (≥75 years), but this difference was less evident among younger patients. Strengths of our study include its large sample size, extensive capture of pertinent confounders, and robust study design, including use of IPTW to balance baseline characteristics between patients who initiated treatment with each agent.

Prior data comparing enzalutamide and abiraterone in the setting of mCSPC have been limited. Although clinical trials have established that both enzalutamide and abiraterone combined with ADT offer benefit over ADT alone for treating mCSPC,^3,28,29^ no trials have compared enzalutamide versus abiraterone, and it has remained unclear whether enzalutamide or abiraterone is superior in the mCSPC setting. Indirect comparisons have been attempted using meta-analysis of clinical trials, but with conflicting and nondefinitive results. A network meta-analysis comparing clinical trials for enzalutamide (ARCHES^1^ and ENZAMET^30^) and abiraterone (LATITUDE^31^ and STAMPEDE^32,33^) in the mCSPC setting found similar outcomes for both ARPI compounds, with both more effective than docetaxel.^12^ In contrast, a different network meta-analysis of mCSPC clinical trials found that abiraterone had superior OS to enzalutamide, but enzalutamide had superior radiographic progression-free survival; however, the study noted that longer follow-up for OS was needed.^13^ A meta-analysis of eight different trials, including those with or without concurrent docetaxel treatment, revealed an OS advantage of enzalutamide compared with abiraterone in patients with low-volume mCSPC.^14^ Finally, a handful of smaller retrospective studies have been published based on cohorts in China^15^ (18 patients treated with abiraterone, 21 patients with enzalutamide), Pakistan^16^ (60 with abiraterone, 20 with enzalutamide), and Japan^17^ (297 with abiraterone, 127 with enzalutamide), but these have been limited by much smaller sample size, shorter length of follow-up, and/or study design.

Given that both drugs independently improve PCS, combining them might be expected to offer additional benefit. However, trials in metastatic castration-resistant prostate cancer found no or limited efficacy from combining abiraterone and enzalutamide, with significantly higher toxicities.^34-36^ Meta-analysis of clinical trial data suggests similar results in the mCSPC setting, with no added benefit from combination therapy and increased toxicity.^37^ One possible explanation is that enzalutamide induces abiraterone metabolism.^34^ Sequential strategies may be more promising; studies suggest that abiraterone followed by enzalutamide leads to better outcomes in terms of OS, progression-free survival, and PSA response rates, at least in docetaxel-naïve metastatic castration-resistant prostate cancer.^38,39^ Whether sequential strategies offer similar benefits in mCSPC warrants further investigation.

Our finding that enzalutamide is superior among older patients (≥75 years) but not younger patients is notable and merits further investigation. Our finding is consistent with real-world data in the mCRPC setting, which found that enzalutamide had greater benefit among older Medicare patients (≥75 years) but not younger patients.^40^ Our finding is also consistent with a suggestive indirect comparison using meta-analysis of mCSPC and nonmetastatic castrate-resistant prostate cancer clinical trials, which found no benefit to adding abiraterone treatments for older patients, but did find a benefit for non-abiraterone ARPIs including enzalutamide.^41^

Regarding etiology, the fact that we observed a benefit in OS and TTS but not PCS suggests that the advantage for enzalutamide in older patients could be due to a lower toxicity profile for enzalutamide compared with abiraterone. Notably, abiraterone requires concomitant corticosteroid administration to prevent mineralocorticoid excess,^42^ and chronic steroid use in older patients is associated with increased cardiovascular risk, worsening glycemic control, and immunosuppression.^43-46^ Additionally, abiraterone has been associated with higher rates of cardiovascular events and hepatotoxicity compared with enzalutamide in pharmacovigilance and real-world studies.^5-8^ Older patients with preexisting cardiovascular disease, diabetes, or hepatic impairment may be particularly susceptible to these effects. Supporting this tolerability hypothesis, a recent multicenter Japanese study comparing ARPIs in mCSPC found that enzalutamide had a significantly lower treatment discontinuation rate due to reasons other than disease progression compared with abiraterone (10% *v* 20%, *P* = .013).^17^ Further studies are needed to definitively establish whether these tolerability differences explain the age-related differential outcomes we observed.

Despite its strengths, our study has limitations. We adjusted for many confounders using IPTW, but IPTW can only balance measured covariates and cannot directly adjust for unmeasured confounders such as tumor volume, Gleason score, and metastatic burden. However, both abiraterone and enzalutamide are approved for mCSPC without restriction by disease volume or Gleason score, and clinical guidelines do not recommend one over the other based on these factors.^47^ In contrast, the differing toxicity profiles of the two agents provide a plausible rationale for treatment selection based on comorbidities such as cardiovascular disease and diabetes, which we measured and balanced. Nonetheless, residual confounding from unmeasured variables cannot be excluded and may influence our findings, including subgroup analyses. We were also unable to confirm concomitant prednisone use among patients receiving abiraterone, although this is standard clinical practice. In addition, fewer patients were at risk of PCS compared with OS in later follow-up periods because the data source for cause of death (VA Mortality Data Repository) lags behind overall mortality data, resulting in earlier censoring for the PCS outcome. Finally, the burden of comorbidity is overall high among Veterans, so although we balanced comorbidities across groups in the cohort, results could differ from those in less comorbid populations.

Although beyond the scope of the current study, outcomes among chemotherapy-exposed patients, a distinct population we excluded due to limited sample size, are of interest for future investigation. Additionally, meta-analyses incorporating international real-world data may provide further validation of our findings.

In conclusion, in this cohort study of patients with mCSPC, we found that enzalutamide and abiraterone had similar treatment outcomes. There was a benefit for enzalutamide among older patients (≥75 years), but this was less evident among younger patients. The findings of this study with real-world data from the largest integrated health care system in the United States may provide guidance for selecting treatments for mCSPC.
